# The Potential and Prospects of Marine Drugs in Intervening Nerve–Tumor Crosstalk

**DOI:** 10.3390/md24060219

**Published:** 2026-06-17

**Authors:** Dan Zhao, Ruiling Xu, Xinyan Xu, Jingxuan Tan, Zhili Zeng, Xirenayi Kanji, Xincan Li, Junchi Hu, Shuai Wang, Yongjun Dang

**Affiliations:** 1 Basic Medicine Research and Innovation Center for Novel Target and Therapeutic Intervention, College of Pharmacy, Chongqing Medical University, Ministry of Education, Chongqing 400016, China; dan_zhao93@163.com; 2 The First Clinical College, Chongqing Medical University, Chongqing 400016, China; ruilingx@163.com (R.X.); 2023221022@stu.cqmu.edu.cn (X.X.); t1590135168@gmail.com (J.T.); 3 College of Pharmacy, Chongqing Medical University, Chongqing 400016, China; 2025222835@stu.cqmu.edu.cn (Z.Z.); 19999415338@163.com (X.K.); xincanli@cqmu.edu.cn (X.L.); 4 Basic Medicine Research and Innovation Center for Novel Target and Therapeutic Intervention, The Second Affiliated Hospital of Chongqing Medical University, Ministry of Education, Chongqing 400010, China

**Keywords:** marine drugs, nerve–tumor crosstalk, cancer pain, oncotherapy

## Abstract

The bidirectional crosstalk between the nervous system and tumors has emerged as a transformative new frontier in both precision oncotherapy and mechanism-driven cancer pain management. Marine natural products with inherent neuroactive properties exhibit unparalleled intervention advantages for targeting this complex pathophysiological axis. Herein, we systematically and prospectively dissect the multi-layered bidirectional communication between the nervous system and malignancies, comprehensively summarize the pivotal contributions of marine-derived bioactive molecules to advances in neuroscience and antitumor therapeutics, and finally provide an outlook on and a call for integrated, interdisciplinary collaboration to enable transformative breakthroughs in the development of marine neuropharmacological agents targeting the nerve–tumor crosstalk axis.

## 1. Introduction

The nervous system and malignant tumors engage in a sophisticated, multidimensional bidirectional crosstalk that profoundly shapes cancer initiation, progression, metastasis, and associated clinical morbidities such as cancer pain [1,2] This interaction extends beyond passive structural proximity to active molecular signaling, wherein nerves modulate tumor behavior while tumors actively remodel neural architecture and function within the tumor microenvironment (TME) [1,2]. Recent advances in cancer neuroscience have illuminated how this crosstalk integrates autonomic (sympathetic and parasympathetic), sensory, and even central neural circuits with tumor cells, immune components, and stromal elements, creating a self-reinforcing niche that favors malignancy. Understanding these mechanisms is essential not only for elucidating tumor biology but also for identifying therapeutic entry points, particularly for marine-derived bioactive compounds that target specific neural receptors and channels.

## 2. Interactions Between the Nervous System and Cancer

### 2.1. Overview of Bidirectional Crosstalk: Neural Influences on Tumor Growth and Tumor-Driven Neural Remodeling

Peripheral nerves exert context-dependent effects on tumorigenesis through distinct autonomic and sensory pathways. Sympathetic nervous system (SNS) fibers, primarily via norepinephrine (NE) release, promote tumor initiation and early progression across multiple malignancies, including prostate [3], breast [4], and pancreatic cancers [5]. NE binds β2-adrenergic receptors (ADRB2) on tumor cells, activating downstream MAPK/ERK and PI3K/AKT/mTOR cascades that enhance proliferation, angiogenesis, epithelial–mesenchymal transition (EMT), and stress-induced survival [6,7]. Clinical correlations show elevated intratumoral SNS density predicts poor prognosis, while chemical sympathectomy or β-blockers attenuate growth in preclinical models [3]. In contrast, parasympathetic nervous system (PSNS) effects are tumor-type specific. In prostate and gastric cancers, acetylcholine (ACh) acting on muscarinic receptors (e.g., CHRM1, CHRM3) drives migration and metastasis [3,8]; however, in certain breast cancer subtypes, PSNS signaling exerts inhibitory effects [9]. The vagus nerve further amplifies this through a tumor-brain axis: tumor-derived factors activate vagal afferents, triggering brainstem sympathetic outflow that reinforces immune suppression [10].

Complementing the autonomic pathways, sensory nerves—particularly peptidergic nociceptors—exert direct pro-tumorigenic effects through neuropeptide release and structural support [11]. These fibers release neuropeptides, such as calcitonin gene-related peptide (CGRP) and substance P, which directly modulate the tumor microenvironment (TME) to favor survival and immune evasion [12,13]. Furthermore, existing sensory nerve fibers serve as physical conduits for perineural invasion (PNI). By providing a nutrient-rich, low-resistance microenvironment along their sheaths, these nerves actively facilitate tumor cell migration, local invasion, and the establishment of metastatic routes [14].

Reciprocally, tumors actively remodel their surrounding neural landscape to sustain and expand this supportive signaling. Cancer cells secrete a diverse repertoire of neurotrophic factors (e.g., NGF, GDNF, BDNF), axon-guidance molecules (e.g., semaphorins, netrins, ephrins), and exosome-packaged signals that drive cancer-associated neurogenesis—the de novo sprouting of nerve fibers into the tumor bed [8,15]. This remodeling process recruits Schwann cells, which adopt a “repair-like” phenotype to guide axonogenesis and further assist in tumor migration [16]. Tumors can also induce the systemic recruitment of neural progenitors from the subventricular zone, which cross the blood–brain barrier to infiltrate the TME and differentiate into adrenergic neurons [17]. Remarkably, tumor-derived signals can even induce the transdifferentiation of existing sensory nerves into an adrenergic phenotype [18]. Together, these bidirectional processes establish a robust ‘neural-tumor ecosystem’ driven by a positive feedback loop: autonomic and sensory nerves provide both biochemical survival signals and physical metastatic conduits, while tumors actively orchestrate neural remodeling and recruitment to perpetuate this supportive network [2]. The molecular underpinnings of this crosstalk converge on specific receptors and signaling axes, which are detailed next and serve as direct targets for marine-derived modulators.

### 2.2. Receptors and Bidirectional Signaling Axes

At the molecular level, bidirectional nerve–tumor crosstalk is orchestrated by a diverse array of ligand-gated ion channels and G-protein-coupled receptors (GPCRs) co-expressed on tumor cells, immune infiltrates, and neural elements, facilitating rapid and continuous reciprocal signaling [19,20]. Nicotinic acetylcholine receptors (nAChRs)—particularly the α7 and α9 subtypes—exemplify this dynamic. Expressed across tumor cells, tumor-associated macrophages, and nerve terminals, nAChRs respond dynamically to acetylcholine (ACh) derived from parasympathetic efferents or synthesized autonomously by the tumor [21]. Receptor activation induces intracellular Ca^2+^ influx, subsequently stimulating the MAPK/ERK, PI3K/AKT/mTOR, and JAK/STAT cascades [22,23]. These pathways collectively drive tumor proliferation, epithelial–mesenchymal transition (EMT), angiogenesis, and anti-apoptotic survival [24,25,26]. Crucially, tumors frequently upregulate nAChR expression while synthesizing endogenous ACh, establishing robust autocrine loops that sustain protumorigenic signaling independently of systemic neural input [21].

The calcitonin gene-related peptide (CGRP) and its receptor complex (CLR/RAMP1) constitute a pivotal sensory-tumor axis. Peptidergic nociceptors release abundant CGRP, which binds CLR/RAMP1 on tumor cells and the local endothelium. This binding elevates intracellular cAMP and activates PKA, thereby promoting cellular proliferation, migration, and local vascular permeability [27]. In gastrointestinal and oral cancers, this axis accelerates neoplastic progression while actively orchestrating immune suppression—specifically by inhibiting CD8^+^ T-cell infiltration and polarizing macrophages toward an immunosuppressive M2 phenotype [28]. Furthermore, tumor-derived factors stimulate reciprocal CGRP production, which induces axonogenesis and central sensitization [29]. This establishes a “neural addiction” paradigm, closing a pro-tumor, pro-pain loop that has recently been identified as a dominant driver in head and neck squamous cell carcinoma (HNSCC) and gastrointestinal malignancies [30].

Transient receptor potential vanilloid 1 (TRPV1) channels, predominantly localized on sensory nociceptors, serve as key integrators of tumor-derived microenvironmental stressors, including severe acidosis, extracellular ATP, and proteolytic enzymes. Channel activation depolarizes these primary afferents, triggering the localized release of Substance P and CGRP. This secretion exacerbates neurogenic inflammation, facilitates perineural invasion (PNI), and promotes extracellular matrix remodeling [31]. In aggressive models, such as breast cancer bone metastasis and oral squamous cell carcinoma, TRPV1 is further sensitized via crosstalk with protease-activated receptor 2 (PAR2), markedly enhancing metastatic colonization and hyperalgesia [32,33]. Additionally, TRPV1 expression on tumor cells enables direct responsiveness to metabolic stress, thereby enhancing cellular motility [34]. Consequently, genetic ablation or pharmacological inhibition of TRPV1 concurrently mitigates cancer-induced pain and restricts tumor burden.

Purinergic signaling introduces another critical layer of bidirectional communication, primarily mediated by P2X7 receptors. These ATP-gated cation channels, expressed on neural elements, immune cells, and tumor cells, respond to the high concentrations of extracellular ATP (eATP) characteristic of the necrotic tumor microenvironment (TME) [35]. Sustained P2X7 activation induces significant Ca^2+^ and Na^+^ influx, which triggers the assembly of the NLRP3 inflammasome and the subsequent release of pro-inflammatory cytokines, notably IL-1β and IL-18 [36]. This purinergic cascade fuels chronic, low-grade neuroinflammation, recruits myeloid-derived suppressor cells to facilitate immune escape, and further amplifies local TME acidification, establishing an inflammatory milieu highly conducive to tumor progression [37,38].

Beyond ligand-gated receptors, voltage-gated and acid-sensing ion channels tightly regulate the excitability of this neuro-neoplastic interface [39]. The voltage-gated sodium channel Na_V_1.7, highly expressed in nociceptive neurons, is essential for action potential generation and continuous neurotransmitter release. Tumor-induced neural hyperexcitability upregulates Na_V_1.7, thereby sustaining intractable cancer pain and the persistent secretion of protumorigenic neuropeptides [40]. Concurrently, voltage-gated potassium channels (e.g., the K_V_1.x family) regulate the resting membrane potential of both cell types; their dysregulation by TME hypoxia alters cell cycle dynamics and tumor migration [41]. Additionally, acid-sensing ion channels (ASIC3) detect aggressive metabolic acidification, further exacerbating the pathophysiology of bone metastasis and its associated pain [42].

Collectively, these diverse receptor systems do not operate in isolation but form integrated, closed-loop circuits. Neural ligands activate potent oncogenic cascades within tumor cells, which reciprocally secrete neurotrophins, proteases, and autocrine factors that reinforce sensory sensitization and localized innervation. Elucidating these bidirectional signaling axes provides precise molecular targets to simultaneously dismantle tumor progression, reverse immune evasion, and alleviate cancer-associated pain.

### 2.3. Pathological Outcomes

The multidimensional neuro-neoplastic crosstalk culminates in two intertwined pathological outcomes: malignant tumor progression and intractable cancer pain. Mechanistically, tumors co-opt nerve fibers for physical metastasis via perineural invasion (PNI) and actively degrade peripheral myelin, resulting in cancer-induced nerve injury (CINI) [14]. This chronic nerve injury triggers inflammatory cascades that recruit immunosuppressive cells (such as MDSCs and TAMs), exhausting effector T cells and driving severe resistance to immunotherapies like anti-PD-1 [43,44]. Concurrently, under sustained therapeutic pressure, tumor cells hijack neural paracrine signals to undergo neuroendocrine transdifferentiation (NEtD), rewiring their cellular lineage to acquire robust resistance to targeted and endocrine therapies [45]. Thus, the neural niche acts as a fundamental catalyst for immune evasion and therapeutic failure.

**Figure 1:**
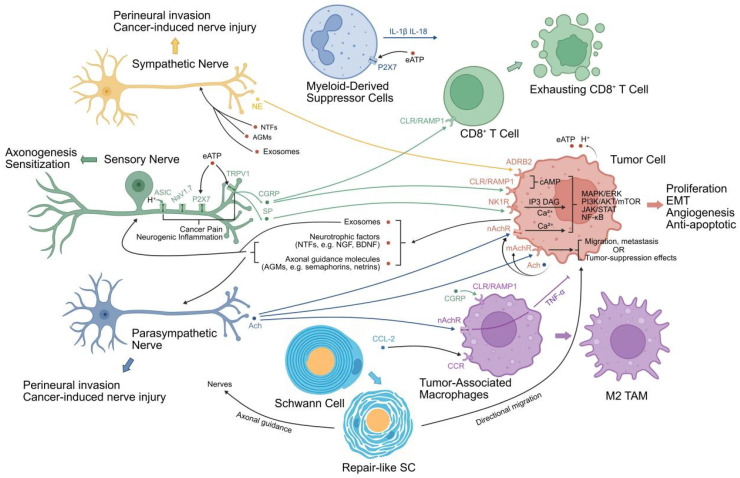
The Multidimensional Bidirectional Crosstalk Within the Nerve–tumor Crosstalk. The nervous system and malignant tumors engage in a sophisticated, multidimensional bidirectional crosstalk. Autonomic and sensory nerves provide biochemical survival signals to drive tumor progression and immune evasion. Reciprocally, tumor-derived factors and microenvironmental stressors actively orchestrate neural remodeling, axonogenesis, and intractable cancer pain, creating a self-reinforcing pathological niche. Created with BioGDP.com [47].

Intrinsically coupled with this malignant progression is the clinical symptom of refractory cancer pain, which functions as a pathological accelerator rather than a passive byproduct. Microenvironmental stressors persistently activate ion channels (e.g., TRPV1, ASICs) on sensory neurons, eliciting severe pain and neuronal sensitization [39]. Crucially, these hypersensitive nociceptive nerves retrogradely release neuropeptides (such as CGRP) into the microenvironment, which reactivate potent oncogenic pathways in tumor cells. This establishes a vicious “pain-cancer” positive feedback loop—often termed “neural addiction”—where tumor growth induces painful nerve injury, and the resulting pain sensitization reciprocally fuels tumor proliferation [30]. Disrupting this intractable cycle to achieve simultaneous analgesia and tumor suppression remains a formidable clinical challenge. Consequently, discovering modulators that target specific neural receptors (e.g., nAChRs, CGRP receptors, and TRPV1) is exceptionally urgent. Marine natural products, distinguished by their unique molecular scaffolds and high target affinities, provide an unparalleled strategic reservoir for developing next-generation, dual-functional therapeutics capable of intervening in this neurogenic pain and tumor progression axis [46] (Figure 1).

## 3. Landmark Marine-Derived Active Molecules Targeting Neuronal Receptors in Oncology

### 3.1. Active Molecules Targeting Nicotinic Acetylcholine Receptors (nAChRs)

#### 3.1.1. α-Conotoxins

**Figure 2:**
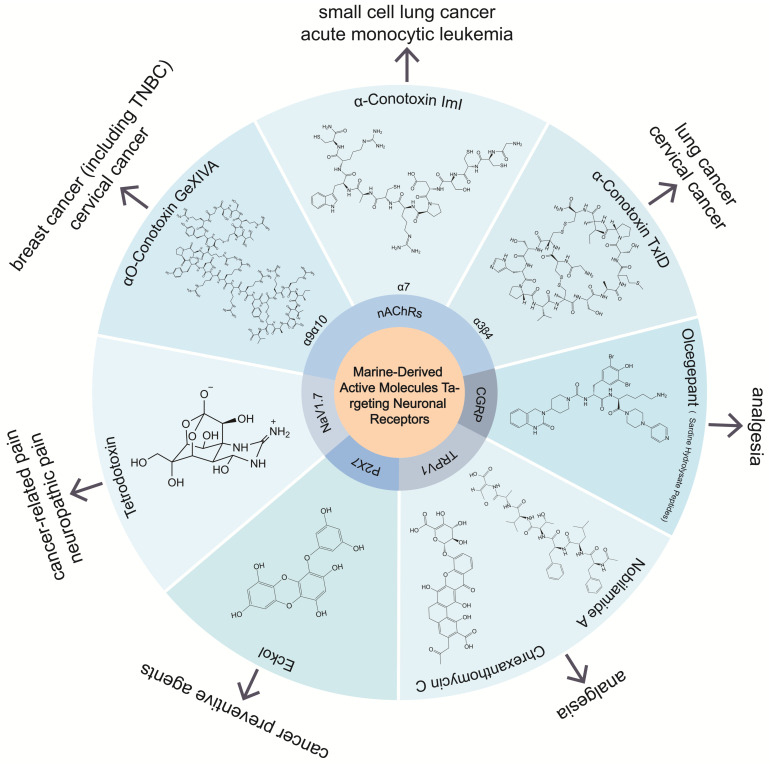
Iconic Marine-Derived Neuroreceptor-Targeting Bioactive Molecules in Oncology.

α-Conotoxins are small peptides derived from the venom of marine cone snails (*Conus* spp.) that can efficiently and selectively block different subtypes of nicotinic acetylcholine receptors (nAChRs). Since the 1980s, these toxins have not only played an important role in neuropharmacology, but in recent years, they have also been widely used to explore the functions of nAChRs in cancer development and progression, showing great potential as anticancer lead compounds (Figure 2).

(1) Targeting the α7 Subtype

Early related studies have confirmed that α-conotoxin ImI, an α7 receptor antagonist, can effectively inhibit the cell proliferation process induced by nicotine or cytisine in small cell lung cancer cell lines (GLCS, NCI-N592, NCI-H69) [48,49]. In the acute monocytic leukemia THP-1 cell model, ImI can upregulate pro-inflammatory factors TNF-α and IL-8 by inhibiting the activity of α7 receptors [50]. Another antagonist with α7 receptor selectivity, ArIB[L11D16], exerts an inhibitory effect on the proliferation of A549 lung cancer cells via the p-Akt signaling pathway [51].

(2) Targeting the α3β4 Subtype

α-Conotoxin AuIB is an α3β4 receptor antagonist that can effectively reduce the viability of DMS-53 small cell lung carcinoma cells [52]. TxID is another selective antagonist for the α3β4 receptor; it not only inhibits the growth of A549 and NCI-H1299 lung cancer cells and enhances the inhibitory efficacy of the chemotherapeutic drug adriamycin [53], but also significantly inhibits the proliferation of cervical cancer SiHa and CaSki cells [54].

(3) Targeting the α9α10 Subtype

αO-Conotoxin GeXIVA is a potent blocker of the α9α10 receptor. Related studies have shown that this substance can inhibit the proliferation of 17 different breast cancer cell lines [55]. In MDA-MB-157 breast cancer cells, GeXIVA not only inhibits cell proliferation and induces apoptosis but also decreases cell migration ability [56] In in vivo experiments, GeXIVA at a very low dose (0.1 nmol/mouse) inhibited tumor growth in mice bearing triple-negative breast cancer 4T1 xenografts, primarily by downregulating the AKT-mTOR, STAT3, and NF-κB signaling pathways and inducing caspase-3-dependent apoptosis [57]. In addition, GeXIVA exhibits inhibitory effects on the growth and proliferation of SiHa and CaSki cervical cancer cells [54].

(4) Other α-Conotoxins and Analogs

Cal14.1a, isolated from *Conus californicus*, exhibits cytotoxicity against four lung cancer cell lines: H1299, H1437, H1975, and H661. Its homologous analog, Cal14.1b, primarily shows cytotoxicity against H1299 cells [58,59]. Among them, Cal14.1a exerts its effects through the caspase-3/7 apoptotic pathway, while Cal14.1b achieves its anticancer effects via a non-caspase-dependent mechanism. Furthermore, α-conotoxin RegIIA and especially its highly selective α3β4 antagonist analog RegIIA[A11A12] are predicted to have high anticancer potential [60].

#### 3.1.2. Sea Anemone Peptide Ms11a-3

Ms11a-3 is a 42-membered peptide isolated from the sea anemone *Metridium senile* with an inhibitor cystine knot fold structure. It has been identified as a potent inhibitor of the α9α10 receptor (while also having some effect on α7 and muscle-type nAChRs) and is predicted to possess anticancer activity [61].

#### 3.1.3. Non-Peptide Small Molecule Compounds

(1) Pinnatoxin G

Pinnatoxin G is a cyclic imine neurotoxin derived from the dinoflagellate *Vulcanodinium rugosum*, which binds to muscle-type, α4β2, and α7 nAChR subtypes [62,63]. At a relatively high concentration (5 μM), this toxin exerts cytostatic and cytotoxic effects on multiple cancer cell lines (HT29 colon cancer, LN18 and U373 glioma, MDA-MB-231 breast cancer, and PC3 prostate cancer cells) [64].

(2) Polyalkylpyridinium salts (poly-APS) and their synthetic analogs APS8 and APS7

Poly-APS is derived from the marine sponge *Haliclona (Rhizoneira) sarai* [65]. Its synthetic analog APS8 is a potent non-competitive α7 nAChR antagonist capable of inducing apoptosis and inhibiting tumor cell growth in non-small cell lung cancer (A549, SKMES-1) and HT29 colon adenocarcinoma cells [66,67] APS7 is also an effective nAChR antagonist that can reverse the anti-apoptotic effects of nicotine on A549 human lung adenocarcinoma cells. When encapsulated in gelatin nanoparticles, APS7 can restore or improve the efficacy of the chemotherapeutic drug cisplatin, which is otherwise reduced by nicotine [68].

(3) Rhizochalinin

Rhizochalinin, initially isolated from the marine sponge *Rhizochalina incrustata*, shows micromolar affinity for muscle-type and neuronal α7 nAChRs [69]. It significantly reduces the in vitro viability of several human prostate cancer cell lines (PC-3, DU145, LNCaP, etc.) and the in vivo growth of PC-3 and 22Rv1 tumors, inducing caspase-dependent apoptosis in vivo. However, subsequent studies identified voltage-gated potassium channels as the main molecular target of rhizochalinin, rather than nAChRs [70].

### 3.2. Active Molecules Targeting CGRP Receptors

#### Sardine (*Sardina pilchardus*) Hydrolysate Peptides

Secondary metabolites, including triterpenoids and fucoidan, derived from the brown algae *Sargassum* sp. have been confirmed through in silico screening (molecular docking) studies to target and bind to the CGRP receptor [71]. Their molecular docking binding affinities are close to those of clinical positive control drugs, and their key interacting amino acid residues are highly identical to the controls. These active substances possess the potential to inhibit the CGRP receptor, providing a theoretical basis and candidate directions for the subsequent experimental verification and development of marine-derived analgesic drugs [72].

### 3.3. Active Molecules Targeting the TRPV1 Channel

#### 3.3.1. Kunitz-Type Peptides Derived from the Sea Anemone (*Heteractis crispa*)

Early studies have established TRPV1 as a core integrator of pain and inflammatory stimuli, making it a key target for the development of novel analgesic drugs. Naturally derived peptides have become an important research direction due to their high selectivity.

(1) APHC1

As early as 2008, Andreev et al. isolated and identified the Kunitz-type polypeptide APHC1 from the venom of the tropical sea anemone *Heteractis crispa*. This study demonstrated that APHC1 is the first polypeptide inhibitor targeting TRPV1. Through electrophysiological and animal experiments, its core mechanism of action was elucidated: APHC1 binds to the extracellular domain of TRPV1 with high affinity and partially inhibits capsaicin-induced cation influx via allosteric modulation, rather than directly blocking the pore. Consequently, it significantly suppressed nociception and exerted a clear peripheral analgesic effect in the mouse tail-flick test and capsaicin-induced pain models. This discovery marks a milestone breakthrough in the targeted modulation of the TRPV1 pain pathway by marine-derived polypeptides [73].

(2) APHC3

In 2013, Andreev et al. further conducted a systematic pharmacological evaluation of the Kunitz-type polypeptides APHC1 and APHC3 derived from the sea anemone *Heteractis crispa*. They confirmed that both are partial antagonists of TRPV1, capable of significantly inhibiting capsaicin-activated channel currents. Notably, APHC3 could specifically block TRPV1 activation induced by acidic pH (pH 5.5), whereas APHC1 lacked this effect. In various mouse pain models, both polypeptides produced potent analgesic effects at low doses without causing the side effect of hyperthermia. This provided an important basis for the development of safer TRPV1-targeted analgesic polypeptides [74].

(3) HCRG21

In 2016, Monastyrnaya is covered the Kunitz-type polypeptide HCRG21 from the sea anemone *Heteractis crispa* and confirmed it as the first full polypeptide antagonist of the TRPV1 receptor [75]. Studies revealed that HCRG21 functions through a dual mechanism involving extracellular pore blocking and intracellular regulatory domain binding: on one hand, it binds to the extracellular pore region of TRPV1, forming strong interactions with the channel’s acidic sites via key positively charged residues, thereby compressing the pore diameter to form a hydrophobic seal; on the other hand, it binds to the intracellular regulatory domain of TRPV1, allosterically locking the channel in a closed conformation. Ultimately, this leads to an almost complete inhibition of capsaicin-induced cation influx, providing a novel prototype molecule for marine polypeptide analgesia research [75].

#### 3.3.2. Nobilamides A-H

Nobilamides A-H are the first long-acting, nearly irreversible TRPV1 antagonists discovered from mollusk-associated bacteria. Targeting the pore-loop segment of the TRPV1 channel, Nobilamides A-H bind to the C578, C621, and F660 residues via dehydrobutyrine (Dhb), blocking ion permeability and inhibiting TRPV1 activation by capsaicin, heat, and protons. Both their binding site and mechanism of action differ from those of classical vanilloid-site antagonists, representing a novel mode of TRPV1 blockade and providing a natural template for the design of marine analgesic leads and long-acting antagonists [76] (Figure 2).

#### 3.3.3. Marine Cyclic Guanidine Alkaloids Isolated from the Sponge *Monanchora pulchra*

The cyclic guanidine alkaloids monanchomycalin B and urupocidin A, isolated from the marine sponge *Monanchora pulchra*, selectively inhibit TRPV1, TRPV2, and TRPV3 channels, but are inactive against the TRPA1 receptor. Among them, monanchomycalin B is the most active non-peptide marine-derived TRPV antagonist published to date. Moreover, they are the first reported marine alkaloids affecting the TRPV2 receptor, providing a natural molecular reference for TRPV channel research and the design of marine-derived analgesic and anti-tumor drugs [77].

#### 3.3.4. Chrexanthomycins

Recent studies have discovered a novel family of hexacyclic xanthones (chrexanthomycins) from the metabolites of marine actinobacteria, which can efficiently target the TRPV1 channel and exert analgesic effects. Among them, chrexanthomycin C (cC) and chrexanthomycin F (cF) exhibit significant TRPV1 inhibitory activity. The IC_50_ of cC is as low as 0.9 μM, directly binding to the channel and blocking capsaicin-induced Ca^2+^ influx. Patch-clamp assays confirmed that both compounds directly inhibit TRPV1 channel currents, and in vivo mouse pain models revealed that they effectively suppressed capsaicin-induced hyperalgesia (pain sensation), comparable to the classical TRPV1 inhibitor capsazepine. These compounds possess a high safety profile with no obvious cytotoxicity, providing an important basis for further investigations into marine-derived TRPV1 modulators for pain management [78] (Figure 2).

### 3.4. Active Molecules Targeting P2X7

#### 3.4.1. Phlorotannins

In the early exploration of marine active molecules targeting P2X7, algae-derived phlorotannins demonstrated significant chemopreventive potential. A 2021 study revealed that phlorotannin-rich extracts from brown algae (such as *Ascophyllum nodosum* and *Fucus vesiculosus*) significantly inhibited benzo [a]pyrene-induced P2X7 receptor activation in human lung epithelial cells (A549). Simultaneously, these extracts attenuated F-actin cytoskeleton rearrangement and the downregulation of E-cadherin expression, suggesting a chemopreventive mechanism via interference with P2X7-related pathways. This finding indicates that phlorotannin-rich brown algae extracts hold promise for blocking carcinogenesis and airway toxicity induced by environmental pollutants, potentially serving as marine-derived cancer preventive agents [79] (Figure 2).

#### 3.4.2. Sea Anemone Kunitz-Type Peptides

As research shifted toward more specific biological macromolecules, a 2023 study focused on peptides from the venom of venomous marine animals. Pislyagin and colleagues utilized biotechnological methods to prepare recombinant analogs of sea anemone Kunitz-type peptides (specifically HCRG1 and HCGS1.10). Molecular docking results suggested that peptides such as HCRG1 might exert their inhibitory effects by binding to the extracellular domain of P2X7, thereby obstructing the conformational transition of the channel from a closed to an open state. These recombinant peptides exhibit significant neuroprotective activity, effectively inhibiting P2X7 receptor-mediated calcium influx and membrane pore formation in Neuro-2a cells, thereby alleviating ATP-induced cellular damage. This provides a novel peptide template for the development of drugs treating neuroinflammation and neurodegenerative diseases, marking a stage of precise intervention in protein structure and function for marine-derived P2X7 modulators [80].

### 3.5. Active Molecules Targeting Na_V_1.7

#### 3.5.1. Glycoglycerolipids

Two glycoglycerolipids isolated from the red alga *Halymenia* sp. have been found to inhibit Na_V_1.7. Fluorescence-based thallium flux assays revealed that one of the glycoglycerolipids possesses an IC_50_ of 6.9 μmol/L, while the other one exhibited weaker activity in the same assay. However, electrophysiological evaluation via automated patch-clamp indicated that the latter has a higher affinity for the inactivated state of Na_V_1.7 (Kd = 25.2 μmol/L) compared to the former (Kd = 49.9 μmol/L). Both compounds demonstrate state-dependent inhibition. Furthermore, the former showed weak cytotoxicity against leukemia and colon cancer cells, while the latter induced apoptosis in lymphocytic leukemia cells. This study represents the first report of glycoglycerolipid Na_V_1.7 inhibitors from the Halymenia genus [81].

#### 3.5.2. BDS-1 (Sea Anemone Toxin Peptide)

BDS-1 is a 43-amino acid toxin derived from the Mediterranean sea anemone *Anemonia viridis*, containing three pairs of disulfide bonds. In addition to modulating K_V_3 potassium channels, BDS-1 potently inactivates Na_V_1.7 (with a weaker effect on Na_V_1.3) by shifting the channel’s inactivation kinetics rather than directly blocking the pore. This polypeptide is highly selective and also counteracts amyloid β-peptide-induced neuronal death by inhibiting K_V_3.4. As a selective Na_V_1.7 inactivator, BDS-1 serves as a significant tool for the research and development of analgesic drugs [82].

#### 3.5.3. Tetrodotoxin (TTX)

Tetrodotoxin (TTX) is a non-peptidyl neurotoxin derived from marine animals such as pufferfish, exhibiting nanomolar affinity for TTX-sensitive sodium channels, including Na_V_1.7 (IC_50_ ≈ 2 nmol/L) [83]. In visceral pain models, subcutaneous injection of TTX (1–6 μg/kg) dose-dependently reduced pain-related behaviors induced by intracolonic capsaicin and mustard oil, as well as by intraperitoneal cyclophosphamide. It also reversed referred mechanical hyperalgesia caused by capsaicin and cyclophosphamide. In conditional Na_V_1.7 knockout mice, the analgesic effect of TTX persisted, suggesting that its action is not dependent on the Na_V_1.7 subtype alone [83]. TTX does not cross the blood–brain barrier and does not cause motor coordination impairment at effective doses, demonstrating a favorable safety profile [83,84]. Currently, TTX has entered clinical trials for the treatment of cancer-related pain and chemotherapy-induced neuropathic pain [84] (Figure 2).

Collectively, marine organisms serve as a rich source of bioactive molecules targeting neuronal receptors, particularly nAChRs, TRPV1, P2X7, NaV1.7 and CGRP receptors. These molecules exhibit significant potential in antitumor therapy (e.g., GeXIVA, TxID, APS8) and analgesia (e.g., APHC peptides, nobilamides, chrexanthomycins, BDS-1, TTX), with some having advanced to preclinical or clinical trial stages. These findings provide important lead compounds and design templates for the development of novel antitumor and analgesic agents.

## 4. Approved Marine-Derived Drugs

**Figure 3:**
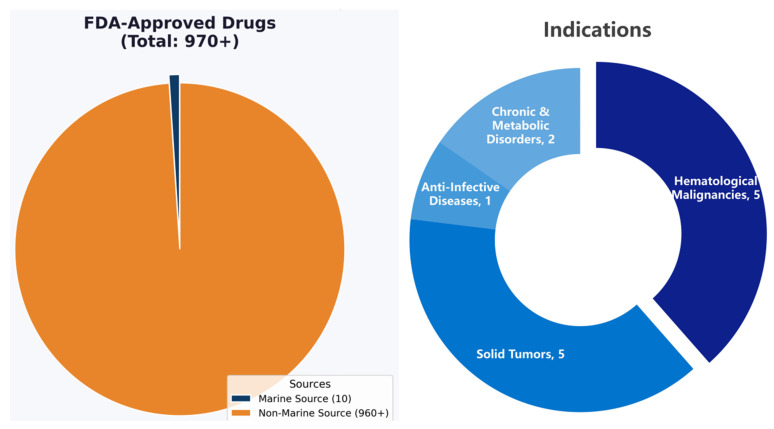
The proportion of drugs derived from marine sources among those approved by the FDA.

Given the differences in light, temperature, salinity, pressure and gas content of marine environment and the vast biodiversity of the oceans, it is estimated that there will be a significant increase in the number of new molecules from marine origin used to deal with cancer. And the facts bear this out: as of 2025, multiple marine-derived drugs have received regulatory approval—including FDA approval—for clinical use (Figure 3). As summarized in Table 1, a detailed review of the FDA database and the literature [85] reveals that approximately ten agents have been approved to date. The first seven entries are agents with approved anticancer indications, followed by three additional marine-derived drugs approved for other therapeutic areas, including antiviral (vidarabine), analgesic (ziconotide), and lipid-lowering (icosapent ethyl) indications.

These include compounds isolated from marine fungi, sponges, and other ocean-derived sources. Three categories of marine-derived agents are distinguished based on their origin: (1) Natural marine products—this refers to compounds directly extracted from marine organisms without chemical modification. Representative examples include vidarabine, fludarabine and cladribine. (2) Marine-derived compounds—these are natural products that have been obtained through total or semi-synthesis, with their chemical structures identical or highly similar to the native marine molecules. This category includes trabectedin, ziconotide and lurbinectedin. (3) Synthetic marine-inspired analogues—these are fully synthetic compounds whose chemical scaffolds are inspired by marine natural products but are structurally distinct from the original leads. Examples include eribulin, pralatrexate, and icosapent ethyl, several of which demonstrate selective antiproliferative activity against tumor cell lines in preclinical models. However, numerous drugs have already been approved for marketing and have been incorporated into clinical treatments, such as Cytarabine, Trabectedin, Lurbinectedin, Eribulin, Pralatrexate, Fludarabine, Cladribine, Vidarabine, Ziconotide, Icosapent ethyl. Since these marine-derived drugs have been approved for clinical use, their safety and efficacy have been systematically evaluated, establishing an acceptable benefit–risk balance in the target patient populations. Therefore, our focus here is to summarize their marine organisms, therapeutic applications, and chemical classes (Table 1 and Table 2).

Level 1: Direct Evidence

Must satisfy at least one of the following criteria: Bidirectional signaling modulation between neurons and tumor cells has been experimentally demonstrated in rigorously validated co-culture systems. The compound has been shown to significantly alter nerve infiltration density or disrupt nerve–tumor signal transmission in established in vivo tumor innervation models. Direct biochemical confirmation of modulatory effects on signaling pathways uniquely implicated in neuron–tumor crosstalk—including but not limited to the CGRP-RAMP1 and NGF-TrkA bidirectional axes—has been obtained.

Level 2: Indirect Evidence

Satisfies only one of the following criteria, with no direct experimental evidence demonstrating modulation of neuron–tumor crosstalk: Acts on well-characterized molecular targets known to mediate neuron–tumor interactions, yet lacks functional validation in co-culture systems or in vivo tumor innervation models. Exhibits both independent anticancer activity and analgesic/neuropharmacological activity, but the mechanistic connection between these dual effects—specifically via neuron–tumor crosstalk—remains unestablished. Modulation of relevant signaling pathways has been confirmed in a single cell type (neurons or tumor cells alone), but not within the context of intercellular neuron–tumor communication.

Level 3: Hypothetical Evidence

Satisfies only one of the following criteria, with neither direct nor indirect functional evidence supporting relevance to neuron–tumor crosstalk: Target binding affinity is predicted exclusively through computational methods (e.g., molecular docking, molecular dynamics simulations, or pharmacophore modeling), without experimental validation. Exhibits only a single isolated pharmacological activity—either anticancer or analgesic—with no demonstrated functional or mechanistic link to the complementary biological domain. Relevant biological activity is observed solely in non-tumor or non-pain disease models, lacking validation in contexts involving tumor innervation or cancer-related neuropathic pain. Potential activity is inferred exclusively from structural homology to known bioactive compounds, without empirical substantiation.

## 5. Marine-Derived Analgesic Drugs Targeting the Nervous System: Marketed Drugs and Preclinical Candidates

### 5.1. Ziconotide for Analgesia

Ziconotide is a synthetic peptide derived from ω-conotoxin MVIIA, a component of the venom of the marine cone snail *Conus magus*. It is the first marine-derived analgesic approved for clinical use. The peptide consists of 25 amino acids and contains six cysteine residues that form three disulfide bonds, which constitute the structural basis required for its pharmacological activity [108]. Ziconotide selectively and specifically blocks N-type voltage-gated calcium channels (Ca_V_2.2) located on presynaptic terminals in the superficial layers of the spinal dorsal horn, thereby inhibiting calcium influx and reducing the release of excitatory nociceptive neurotransmitters such as glutamate and substance P. In this way, it interrupts pain signal transmission [108].

The clinical development of ziconotide has shown a clear expansion from a focus on specific pain syndromes to broader applications in severe pain management. Early studies, as summarized in a 2017 systematic review, primarily evaluated ziconotide as an intrathecal monotherapy for refractory chronic neuropathic pain [109]. With continued improvements in the diagnosis and management of neuropathic pain, as well as in intrathecal drug delivery guidelines [110], more recent systematic reviews and meta-analyses have substantially broadened its clinical scope. These studies have assessed its efficacy and safety in severe chronic pain, including neuropathic pain, cancer-related pain, and nonmalignant chronic pain [111].

Ziconotide therefore holds considerable clinical promise. In the current context of severe pain management, where opioid-related concerns remain prominent, ziconotide not only provides effective analgesia but also avoids the major limitations of conventional opioids, including tolerance and dependence. As such, it represents an important non-opioid option in the analgesic armamentarium [109,111]. Importantly, ziconotide does not bind to opioid receptors and is not associated with addiction or tolerance [109]. A 2017 systematic review and meta-analysis confirmed that intrathecal ziconotide significantly improves refractory neuropathic pain and provides superior pain relief compared with placebo [109]. Subsequent studies have further supported its efficacy in a variety of neuropathic pain conditions, including diabetic peripheral neuropathy, trigeminal neuralgia, phantom limb pain, spinal cord injury-related pain, and complex regional pain syndrome (CRPS) [110,111]. A 2024 review further noted that intrathecal therapy combining ziconotide and morphine may help optimize treatment and reduce adverse events in neuropathic pain and cancer-related pain [110]. In addition, a 2025 meta-analysis including 23 studies and 1531 patients showed that ziconotide significantly reduced pain scores relative to placebo. Observational studies also suggested reductions in opioid consumption ranging from 6.4% to 91.5%, underscoring its dual value as a potent analgesic and an opioid-sparing agent [111].

Despite these advantages, broader clinical use of ziconotide remains limited by challenges related to administration, safety, and evidence quality. Because it cannot cross the blood–brain barrier, ziconotide must be administered intrathecally [109], which restricts its practical use. Adverse events are common, with an overall incidence of 94.9% and a serious adverse event rate of 17.85% in the RCTs included in a recent meta-analysis [111]. These events are mainly neuropsychiatric in nature, including dizziness, nausea, nystagmus, confusion, memory impairment, and ataxia, and their frequency is closely related to the initial dose and titration rate [109,112]. In addition, the overall certainty of evidence remains low to moderate, with substantial heterogeneity across studies, leaving the long-term safety profile and the value of combination therapy insufficiently defined [111]. Other limitations include a narrow therapeutic window, the need for administration via an implanted pump by trained professionals, high cost [109], and limited accessibility. Future work should focus on optimizing dosing and titration strategies and validating clinical utility through higher-quality trials (Table 3).

### 5.2. Sea Anemone Peptide APETx2 for Analgesia

APETx2 is the first marine peptide toxin shown to selectively target ASIC3. Since its isolation from the venom of the sea anemone *Anthopleura elegantissima* in 2004 [113], APETx2 has become an important pharmacological tool and a promising analgesic candidate due to its distinctive molecular structure and target selectivity. APETx2 is a 42-amino-acid basic peptide that adopts a three-stranded antiparallel β-sheet structure stabilized by three disulfide bonds [113]. As a highly selective ASIC3 inhibitor, it rapidly and reversibly suppresses ASIC3-mediated transient peak currents, sustained window currents under pH 7.0 conditions, and alkali-induced human ASIC3 currents, and its inhibitory effect is pH-independent [113,114,115]. At commonly effective concentrations, APETx2 has no meaningful effect on ASIC1a, ASIC1b, or ASIC2a [113]. Only at higher concentrations does it weakly inhibit other ion channels, including Na_V_1.8, Na_V_1.2, and Na_V_1.6 [116,117], suggesting that its analgesic action may involve a limited multi-target component [118].

In animal models, APETx2 exhibits anti-hyperalgesic or analgesic effects in chemical pain, migraine, and bone cancer pain [119,120]. As a non-opioid analgesic candidate, APETx2 may help avoid opioid-associated adverse effects such as addiction and respiratory depression, and thus holds promise for the treatment of neuropathic pain and cancer pain. However, its clinical translation is still constrained by several issues, including uncertain translational efficacy, potential cardiovascular and neurological safety concerns, incompletely elucidated mechanisms of action, and a lack of clinical data (Table 3).

### 5.3. Tetrodotoxin (TTX)

Tetrodotoxin (TTX) is a potent non-peptidic guanidinium neurotoxin [121]. It was originally identified in pufferfish but has since been isolated from a variety of taxa associated with TTX-producing bacteria [122]. TTX selectively blocks voltage-gated sodium channels (VGSCs) and, at therapeutic doses, shows good selectivity for certain isoforms highly expressed in the nervous system, such as Na_V_1.6 and Na_V_1.7 [123], thereby alleviating chronic pain, including neuropathic pain and cancer-related pain. Importantly, it does not affect the TTX-resistant cardiac channel Na_V_1.5 [123], supporting a favorable cardiac safety profile [124].

In neuropathic pain models, TTX has demonstrated robust analgesic effects across multiple rodent systems, attenuating mechanical and thermal hyperalgesia induced by peripheral nerve injury and chemotherapy agents such as paclitaxel and oxaliplatin [125,126]. In a postherpetic neuralgia model, it dose-dependently suppressed pain without causing motor or respiratory adverse effects [121]. For cancer pain, a recent meta-analysis showed that TTX administered at 30 μg twice daily significantly increased the proportion of patients achieving at least a 30% improvement in mean pain intensity. It also provided sustained analgesia, with improvements in quality of life and reductions in opioid consumption reported in some studies [124]. As a non-opioid analgesic candidate, TTX is currently being developed for chemotherapy-induced neuropathic pain and cancer-related pain. A recent randomized, double-blind, placebo-controlled phase I study indicated that TTX was safe and well tolerated, with no clinically relevant cardiac changes and no measurable neuromuscular or respiratory impairment [121,127]. Nevertheless, several challenges remain, including its high toxicity, narrow therapeutic index, uncertainty regarding blood–brain barrier penetration [122], limited long-term safety data, a paucity of preclinical studies on cancer pain [121], and small sample sizes in existing clinical trials [124]. Larger and longer-term clinical studies are still needed to confirm its therapeutic value (Table 3).

## 6. Targeting the Tumor Neural Microenvironment: Therapeutic Applications of Marine-Derived Drugs in Cancer

### 6.1. Application of Marine-Derived Drugs Targeting the Nervous System in Cancer-Related Pain

**Figure 4:**
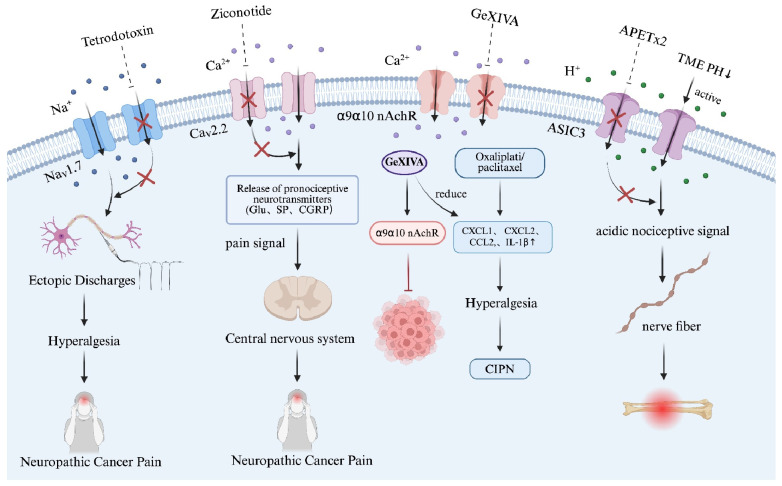
Marine-Derived Drugs Targeting the Nervous System: Applications in Cancer Treatment. Created by BioRender (https://www.biorender.com/, accessed on 13 January 2026).

Pain is among the most prevalent symptoms experienced by cancer patients. The average prevalence of pain across different cancer stages is approximately 40%, escalating to a staggering 74% in advanced stages [128]. Even among patients undergoing active anticancer treatments, the prevalence remains high, ranging from 24% to 60% [129]. The etiology of cancer pain is highly complex, primarily encompassing direct tumor compression or nerve infiltration, bone pain induced by bone metastasis, and nerve injury subsequent to surgery or radiotherapy [130,131]. Herein, we summarize the applications of marine-derived, nervous system-targeting drugs in cancer therapy (Figure 4).

#### 6.1.1. Central Intervention for Refractory Persistent Cancer Pain

In advanced cancer stages, patients frequently endure severe and persistent pain. Prolonged use of opiate analgesics, such as morphine, highly predisposes these patients to tolerance and addiction. Consequently, conventional peripheral administration often fails to achieve optimal efficacy, necessitating direct clinical intervention in the signal transmission of the central nervous system [132]. Ziconotide, a novel non-opioid analgesic synthesized based on ω-conotoxin MVIIA, is the first marine-derived peptide analgesic approved for severe, refractory chronic pain. Unlike opioids, ziconotide produces a potent analgesic effect by selectively blocking N-type voltage-sensitive calcium channels, thereby interrupting neurotransmission from primary nociceptive afferents [94]. Therefore, it does not induce tolerance or addiction. However, owing to its limited ability to cross the blood–brain barrier, ziconotide must be administered via the intrathecal route to achieve optimal analgesia and minimize severe adverse effects. This spinal administration allows ziconotide to reach a maximum local concentration rapidly, facilitating a quick onset of pain relief [94]. In the randomized controlled clinical trials for refractory cancer pain and AIDS-related pain, intrathecal injection of ziconotide significantly reduced patients’ pain intensity, with the mean improvement rate of the Visual Analog Scale for Pain Intensity (VASPI) as high as 53.1%, which was far superior to that of the placebo group (18.1%) [94]. These clinical data substantiate the efficacy of targeting N-type calcium channels for alleviating terminal cancer pain. Furthermore, by effectively blocking nociceptive signal transmission from the periphery, this strategy provides a powerful tool to dissect the contribution of peripheral neuronal activity to the cancer pain experience.

#### 6.1.2. Targeting Ectopic Discharges and Neuropathic Cancer Pain

Neuropathic cancer pain originates from tumor invasion, compression, or nerve damage induced by chemotherapeutic agents [133]. Its core pathological basis lies in the spontaneous ectopic discharges generated by primary sensory neurons, clinically manifesting as burning pain, electric shock-like sensations, and mechanical hypersensitivity [134]. Under pathological conditions such as nerve injury or cancer, certain TTX-sensitive sodium channels (e.g., Na_V_1.3 and Na_V_1.7) on primary sensory neurons are overexpressed. Specifically, the overexpression of Na_V_1.7 leads to increased excitability and ectopic discharges, culminating in hyperalgesia [135]. TTX effectively suppresses the generation of abnormal electrical signals (ectopic discharges) and the conduction of action potentials in the peripheral nervous system by specifically binding to and blocking these sodium channels, thereby cutting off the transmission of nociceptive signals to the central nervous system at the source [121]. Experimental evidence has demonstrated that subcutaneous injection of TTX can safely and effectively alleviate refractory moderate-to-severe cancer pain, with adverse reactions (such as nausea, dizziness, or perioral numbness) being mostly mild and transient [136]. This highlights the potential of TTX as a novel, non-addictive analgesic for cancer pain. However, while TTX primarily acts on the peripheral nervous system due to its poor blood–brain barrier (BBB) permeability, clinical observations of mild and transient central adverse reactions (such as nausea and dizziness) suggest that trace amounts may still cross the BBB or interact with circumventricular organs (e.g., area postrema) that lack tight junctions. This indicates that systemic administration of TTX, although promising, requires careful dose titration to balance analgesia and central side effects.

#### 6.1.3. Targeting Tumor Microenvironment Acidosis-Related Pain

Due to the Warburg effect, the tumor microenvironment (TME) is frequently maintained in a notably acidic state (pH 6.5–5.5) [137]. Protons (H^+^) can activate acid-sensing ion channel 3 (ASIC3) on the terminals of peripheral sensory nerve fibers, thereby inducing pain [138]. ASIC3, a non-selective cation channel activated by acidification, is known to play a pivotal role in modulating inflammatory pain and facilitating nociception in various diseases accompanied by bone pain. APETx2, a potent ASIC3 antagonist derived from sea anemones, has demonstrated excellent targeted efficacy in preclinical studies. In a murine myeloma model, a single local injection of the selective ASIC3 antagonist APETx2 significantly and substantially reduced myeloma bone pain (MMBP) behaviors [42]. Distinct from traditional opioids, the analgesic effect of APETx2 does not rely on central nervous system inhibition. Instead, it acts directly within the TME. APETx2 specifically binds to and locks ASIC3 on primary sensory neurons, blocking this persistent inward current at the nanomolar level, rendering the afferent nerve fibers incapable of sensing the acidic nociceptive signals secreted by the tumor.

#### 6.1.4. Targeting Neuro-Cancer Crosstalk for Pain Alleviation

The nervous system not only supports tumor dissemination via nerve fibers but also regulates tumor growth through neurotransmitters. Among nicotinic acetylcholine receptors (nAChRs), the α9α10 nAChR subtype is not only involved in pain transmission within the peripheral and central nervous systems but has also been implicated in the tumorigenesis, progression, and metastasis of various cancers in preclinical models. GeXIVA, a targeted α9α10 nAChR antagonist, exhibits analgesic effects in various neuropathic pain models, including rat models of chemotherapy-induced peripheral neuropathy (CIPN) induced by oxaliplatin and paclitaxel [139]. Furthermore, it significantly inhibits the proliferation of multiple breast cancer cell lines (such as MDA-MB-231 and MCF-7) in a dose-dependent manner. In murine xenograft models, GeXIVA effectively reduces the volume and weight of tumors, suggesting its dual potential in pain alleviation and tumor suppression [55]. These observations raise the intriguing possibility that GeXIVA might simultaneously interrupt pain signaling and tumor growth signals within the nerve–tumor microenvironment.

### 6.2. Application of Nerve-Targeting Marine-Derived Drugs in Cancer Therapeutics

#### 6.2.1. Targeted Tumor Drug Delivery

Traditional chemotherapeutic agents (e.g., paclitaxel, doxorubicin) face a “double-edged sword” dilemma in clinical application: while killing tumor cells, they frequently induce severe systemic toxicities, such as myelosuppression and chemotherapy-induced peripheral neuropathy (CIPN), due to a lack of tissue specificity. Achieving precise drug delivery by targeting tumor surface receptors can drastically mitigate these toxic side effects. α-Conotoxin ImI, a 12-amino-acid peptide containing two disulfide bonds, is a natural ligand for α7-nAChR characterized by high selectivity, specificity, and potency [140]. Studies have utilized ImI to functionalize PEG-DSPE drug-loaded micelles (ImI-PMs) for paclitaxel encapsulation, facilitating a more rapid and extensive internalization into MCF-7 cells via targeted recognition of α7-nAChR. In mouse models, this approach not only significantly enhanced the tumoricidal efficacy of paclitaxel but also greatly alleviated systemic chemotoxicity, such as weight loss and leukopenia [141]. Furthermore, docetaxel-loaded nanomicelles modified with ImI can specifically penetrate lung cancer cell membranes, driving high drug accumulation precisely at the lung tumor lesions [142].

#### 6.2.2. Blocking Neurogenic Trophic Signals

The nervous system acts not merely as a transmitter of cancer pain, but as an integral component of the tumor microenvironment. Neurotransmitters released by nerve terminals and the expression of ion channels directly regulate tumor proliferation and metastasis. Research indicates that tumor cells can release acetylcholine (ACh), a classic neurotransmitter, to induce epithelial–mesenchymal transition (EMT) and promote intrahepatic cholangiocarcinoma metastasis [143]. Nicotinic acetylcholine receptors (nAChRs, particularly the α7 and α9 subtypes) are highly expressed in breast, lung, and pancreatic cancers. Upon activation by ACh, these receptors trigger downstream MAPK/ERK and PI3K/Akt signaling cascades, profoundly inducing tumor cell proliferation and promoting intra-tumoral microangiogenesis, which provides essential vascular support for tumor growth [144]. Therefore, inhibiting nAChRs to block neurogenic signals and sever the nerve–tumor crosstalk has emerged as a novel strategy to suppress tumor proliferation.

GeXIVA not only exerts a significant anti-invasive effect by blocking EMT but also competitively binds to α9α10 nAChR, thereby antagonizing the cholinergic regulation of tumor cells and inhibiting breast cancer cell proliferation [55]. Similarly, Pinnatoxin G, a marine cyclic imine toxin, acts as an antagonist to nAChRs on the surface of cancer cells. By antagonizing nAChRs, Pinnatoxin G may interfere with cholinergic signaling pathways that support tumor cell survival. Moreover, it induces caspase-dependent apoptosis by disrupting intracellular Ca^2+^ homeostasis, thereby exerting a potent anti-proliferative effect against tumors [145].

### 6.3. Modulation of Neuro-Immune Interactions in the Tumor Microenvironment

The dynamic crosstalk between the nervous system and the tumor microenvironment has emerged as a new frontier in cancer metastasis research. Due to their innate neuroactive properties, marine drugs possess unique advantages in intervening in this complex network. Due to their neuroactive properties, some marine-derived compounds represent useful tools for investigating neuro–immune interactions within the tumor microenvironment. However, evidence demonstrating direct modulation of neuro–immune–tumor crosstalk remains limited.

#### 6.3.1. Neuroprotection and Intervention in Neural Infiltration

Chemotherapeutic agents (e.g., paclitaxel or oxaliplatin) induce toxic damage to peripheral nerve fibers. Neuroprotective strategies can effectively prevent and accelerate recovery from chemotherapy-induced peripheral neuropathy (CIPN). Fucoidan, a classic marine natural polysaccharide widely found in brown algae, upregulates and activates the Gas6/MerTK pathway in macrophages or Schwann cells. By eliminating macrophage-mediated neuroinflammation in the peripheral nervous system and clearing cellular debris, fucoidan preserves the structural integrity of nerve fibers and effectively alleviates neuropathic pain [146]. RgIA4, an engineered analog of RgIA, can prevent neuropathic pain caused by oxaliplatin-induced peripheral neuropathy and lacks activity against GABA_β receptors [147]. RgIA4 also significantly shortens the duration of mechanical allodynia induced by paclitaxel in rats, accelerates the functional recovery of sensory nerves, and maintains these effects long-term even after treatment cessation [148]. These findings suggest that RgIA4 may facilitate recovery of peripheral nerve function in preclinical models, although the underlying mechanisms remain incompletely understood. Furthermore, recent studies have discovered that microalgal metabolites can not only suppress tumor proliferation by inhibiting key survival signaling pathways such as PI3K/Akt/mTOR but also enhance neurotrophic support and promote synaptic remodeling, effectively protecting and repairing damaged neurons [149].

#### 6.3.2. Neuroinflammation and Immune Activation

Local tissue microenvironment damage caused by tumor growth or chemotherapy toxicity stimulates peripheral sensory neurons to release neurogenic inflammatory mediators, the most potent being neuropeptides and substance P. These signaling molecules regulate the activity of innate and adaptive immune cells [150]. The activated immune network, in turn, releases proinflammatory cytokines (e.g., TNF-α, IL-1β), continuously sensitizing peripheral nociceptive nerve endings [151]. Studies have confirmed that α9α10 nAChRs are not only expressed on the sensory nerve endings of the dorsal root ganglion (DRG) [152], but are also widely distributed on the surface of various immune cells, such as macrophages and T cells [153]. RgIA, a marine conopeptide targeting α9α10 nAChRs, can significantly inhibit local macrophage infiltration in mice with colitis and drastically reduce the release of proinflammatory cytokines, thereby effectively reversing severe intestinal mucosal damage [154]. Moreover, research has demonstrated that the analgesic and neuroprotective effects of RgIA4 are not merely due to neural blockade; rather, they fundamentally depend on the involvement of the immune system (especially CD3^+^ T cells), as RgIA4 substantially reduces immune cell infiltration at the sites of nerve injury [155].

Collectively, marine-derived neuroactive compounds have shown promising analgesic, neuroprotective, immunomodulatory, or antitumor activities in preclinical and, in some cases, clinical studies. Nevertheless, in most instances, evidence supports effects on individual components of the neural, immune, or tumor compartments rather than direct demonstration of bidirectional nerve–tumor crosstalk. Future studies employing mechanistic models specifically designed to interrogate neural–tumor interactions will be required to establish causal relationships.

## 7. Outlook: A Call for Interdisciplinary Collaboration Targeting Nerve–Tumor Crosstalk

The dynamic crosstalk between the nervous system and the tumor microenvironment has emerged as a transformative new frontier in both oncotherapeutic development and cancer pain management. Marine natural products, characterized by their inherent neuroactive properties, offer unique and unparalleled advantages for targeting and modulating this intricate regulatory network.

### 7.1. Stage 1: Establishment of Marine Natural Product Libraries and High-Throughput/High-Content Screening Platforms

While ongoing preclinical and clinical studies have extensively explored the bioactivities of diverse marine metabolites, the translational path from marine natural products to clinical use remains hindered by multiple critical bottlenecks. Chief among these is the low yield and prohibitive cost associated with the direct extraction of bioactive compounds from marine sources. To address this challenge, the establishment of standardized, well-annotated marine natural product compound libraries is an urgent and foundational priority. Leveraging automated high-throughput and high-content screening platforms enables the rapid identification of bioactive compounds with target-specific properties from tens of thousands of marine-derived molecules [156]. This approach not only supports the sustainable utilization of marine biological resources but also facilitates iterative, multi-target screening campaigns.

### 7.2. Stage 2: Discovery of Dual- and Multi-Functional Marine-Derived Therapeutics

Marine natural products have garnered widespread research attention owing to their structurally diverse scaffolds and unique, target-specific biological activities. Notably, dual-functional marine-derived agents, exemplified by conotoxin GeXIVA, enable the implementation of a two-pronged therapeutic strategy: simultaneous disruption of nerve–tumor crosstalk and alleviation of cancer pain [55]. Likewise, multi-functional marine molecules such as conotoxin RgIA4 [148], which exhibits both neuroprotective and immune-activating properties, hold substantial translational potential in multiple therapeutic areas, including neuroinflammation management and tumor immunotherapy.

### 7.3. Stage 3: Development of Diversified Clinical Translation Strategies

Potent, target-selective marine-derived molecules (e.g., monomethyl auristatin E, MMAE) are already validated as cytotoxic payloads for the construction of antibody–drug conjugates (ADCs) [157]. Beyond this established application, marine-derived peptides with targeted analgesic and neurotropic properties hold great promise as targeting moieties for the development of peptide–drug conjugates (PDCs). Furthermore, multiple cutting-edge technologies can be harnessed to advance the clinical translation of marine natural products. For instance, bioorthogonal activation strategies can be applied to achieve spatiotemporally controlled release of marine-derived bioactive molecules, and prodrug engineering approaches can be exploited to optimize the pharmacokinetic and pharmacodynamic properties of marine metabolites. Meanwhile, given the inherent neuro-targeting capability and high target affinity of marine-derived molecules, their combination with ultra-high-resolution imaging techniques—including stimulated emission depletion (STED) microscopy and fluorescence lifetime imaging microscopy (FLIM)—enables the dynamic, high-precision capture of signaling events underlying nerve–tumor crosstalk. This integrated approach holds the potential to map nerve–tumor interactions with unprecedented spatiotemporal resolution.

### 7.4. Current Obstacles in Marine Natural Product Drug Discovery and Development

Despite the immense chemical diversity and promising bioactives of marine natural products (MNPs), their translation into clinically approved drugs faces several significant obstacles. Compared with terrestrial natural products and synthetic compounds, marine-derived drugs candidates encounter unique challenges that limit their development and clinical translation. MNPs often possess highly complex polycyclic structures with multiple stereocenters, making chemical synthesis extremely challenging and costly, a difficulty that is further compounded by the great variety of marine organisms, which makes it hard to give recommendations for a one-fits-all solution for the extraction, purification and identification of marine peptides [158]. Additionally, many marine-derived bioactive molecules exhibit unfavorable pharmacokinetic properties, including low oral bioavailability and rapid metabolism [159]. For indications targeting the central nervous system, many MNPs fail to achieve therapeutic concentrations in the brain despite potent in vitro activity due to poor blood-brain permeability [160]. Collectively, these factors hinder the clinical translation of numerous marine natural products. And novel formulation strategies, such as nanoparticle encapsulation [161] or lipid-based delivery systems, are required to improve their bioavailability.

### 7.5. Expectation

**Figure 5:**
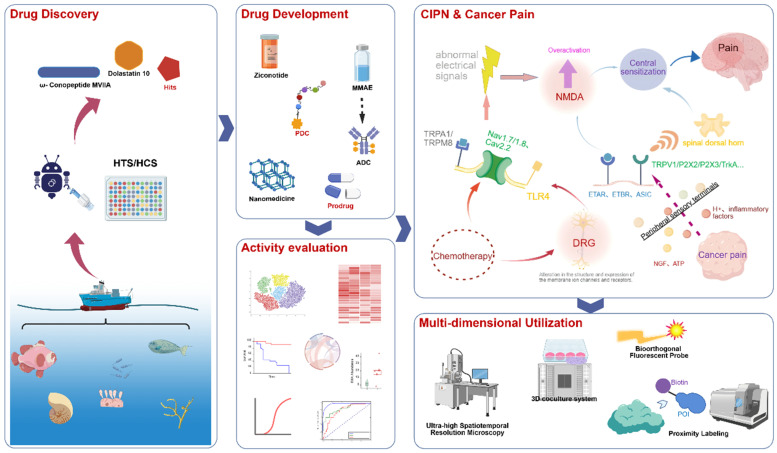
Looking forward to the interdisciplinary integration and translational application of marine drugs in cancer neuroscience research. Created with BioGDP.com (https://biogdp.com/zh, accessed on 13 January 2026) [47].

Collectively, unlocking the full therapeutic potential of marine natural products for targeting nerve–tumor crosstalk cannot be achieved through siloed research efforts. It requires interdisciplinary collaboration that bridges oncology and neuroscience, alongside integrated contributions from chemical biologists, pharmacologists, marine natural product chemists, and experts in high-throughput screening technology. Only through such concerted, cross-disciplinary efforts can we realize the diversified and efficient clinical translation of marine-derived therapeutics, and ultimately develop transformative strategies for the precise modulation of Nerve–Tumor Crosstalk (Figure 5).

## 8. Literature Search and Selection Methodology

Given the rapid development of this interdisciplinary field and the lack of a comprehensive synthesis of current knowledge, we conducted a narrative review to summarize the evidence for marine-derived compounds as modulators of nerve–tumor crosstalk and their potential applications in cancer pain management.

A comprehensive literature search was performed in four major electronic databases: **PubMed/MEDLINE, Web of Science Core Collection, Scopus, and Embase**. The search was conducted from the inception of each database to **[30 April 2026]** to capture all relevant publications. No language restrictions were applied. The search strategy combined **Medical Subject Headings (MeSH) terms** and free-text keywords tailored to the syntax of each database.

The core search terms included combinations of:

(1) Marine-derived compounds: “marine natural products”, “marine drugs”, “marine secondary metabolites”, “marine alkaloids”, “marine peptides”.
(2) Nerve–tumor crosstalk: “nerve–tumor interaction”, “neuro-tumor crosstalk”, “tumor innervation”, “cancer neuroscience”.
(3) Related mechanisms: “neurogenesis”, “axonogenesis”, “neurotransmitters”, “cancer pain”, “tumor microenvironment”.

Studies were included if they met all of the following criteria:

(1) Study type: Original research articles (in vitro cell culture experiments, in vivo animal studies, randomized controlled trials, cohort studies) and high-quality systematic reviews/meta-analyses.
(2) Study content: Directly investigated marine-derived compounds and their effects on nerve–tumor crosstalk, or provided relevant mechanistic insights into marine compounds’ neuropharmacological, analgesic, or anticancer activities.
(3) Publication status: Published in peer-reviewed journals.
(4) Availability: Full text was accessible.

## Figures and Tables

**Table 1:** Marine-derived drugs (FDA approval).

	Structure	Related Source	Indications	Classification of Application Potential	Approval Year	Reference
Cytarabine	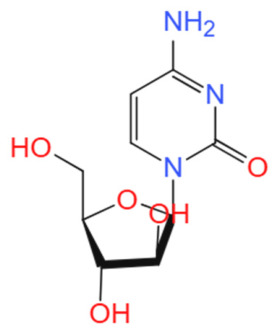	Caribbean sponge (*Cryptotethya crypta*)	Acute myeloid leukemia (AML), Acute lymphoblastic leukemia (ALL)	Level 3	1969	[86]
Trabectedin	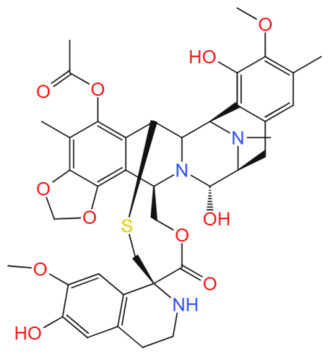	Tunicate (*Ecteinascidia turbinata*)	Soft tissue sarcoma, Ovarian cancer	Level 3	2015	[87]
Lurbinectedin	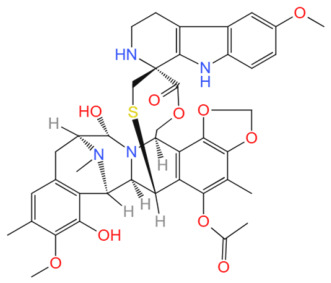	Tunicate (*Ecteinascidia turbinata*)	Small cell lung cancer (SCLC)	Level 3	2020	[88]
Eribulin	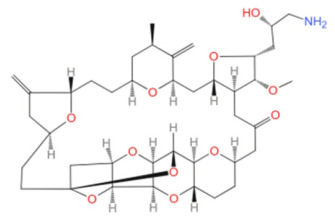	Sponge (*Halichondria okadai*)	Metastatic breast cancer, Liposarcoma	Level 3	2010	[89]
Pralatrexate	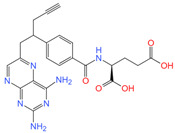	Marine actinomycete (*Streptomyces* sp.)	Peripheral T-cell lymphoma	Level 3	2009	[90]
Fludarabine	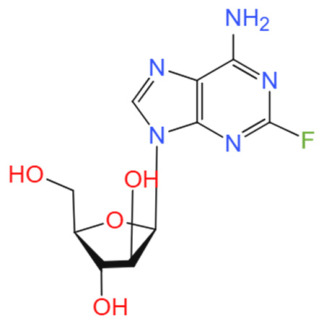	Caribbean sponge (*Cryptotethya crypta*)	Chronic lymphocytic leukemia (CLL)	Level 3	1991	[91]
Cladribine	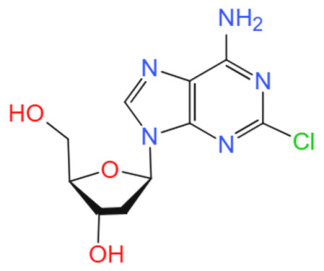	Caribbean sponge (*Cryptotethya crypta*)	Hairy cell leukemia (HCL)	Level 3	1993	[92]
Vidarabine	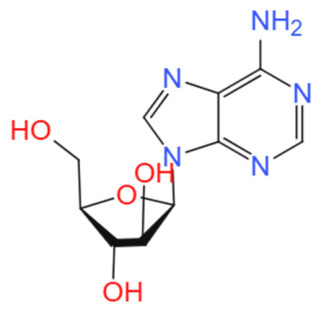	Caribbean sponge (*Cryptotethya crypta*)	Herpes simplex keratitis	Level 3	1976	[93]
Ziconotide	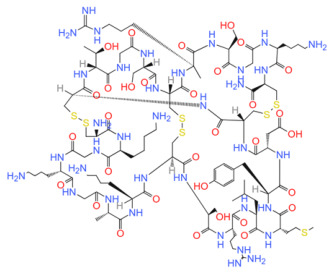	Cone snail (*Conus magus*)	Severe chronic pain	Level 2	2004	[94]
Icosapent ethyl	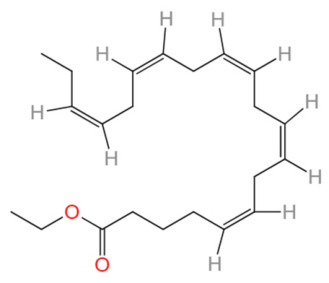	Deep-sea fish (Omega-3 ethyl ester)	Severe hypertriglyceridemia	Level 3	2012	[95]

A three-tier classification system is established based on each drug’s pharmacological characteristics and/or approved indications and its emerging therapeutic potential in neurology and/or oncology, enabling a structured assessment of their clinical and translational value.

**Table 2:** Marine-derived drugs (Investigational drugs).

Drug Name	Related Source	Indications	Classification of Application Potential	Approval Status	Reference
Aplidin	Tunicate (*Aplidium albicans*)	Multiple myeloma	Level 3	Limited Marketed (AU)/Phase III	[96]
Marizomib	Marine bacterium (*Salinispora tropica*)	Glioblastoma multiforme, Multiple myeloma	Level 3	Phase III	[97]
Plinabulin	Marine fungus (*Aspergillus* sp.)	Non-small cell lung cancer (CIN prevention)	Level 3	Phase III/NDA Pending	[98]
Squalamine	Dogfish shark (*Squalus acanthias*)	Ovarian cancer	Level 3	Phase II/III	[99]
E7070	Sponge (*Halichondria okadai*)	Non-small cell lung cancer, Solid tumors	Level 3	Phase II/III	[100]
Bryostatin 1	Bryozoan (*Bugula neritina*)	Alzheimer’s disease, Cancer	Level 3	Phase II	[101]
Spisulosine	Surf clam (*Spisula polynyma*)	Advanced solid tumors	Level 3	Phase II	[102]
KRN5500	Sponge (*Halichondria okadai*)	Cancer pain, Neuropathic pain	Level 2	Phase II	[103]
Soblidotin	Sponge (*Callyspongia truncata*)	Soft tissue sarcoma, Solid tumors	Level 3	Phase II	[104]
BG136	Brown algae (*Sargassum* sp.)	Advanced solid tumors, Advanced colorectal cancer	Level 3	Phase I (NMPA)	[105]
DZNep	Sponge (*Jaspis* sp.)	Acute myeloid leukemia, Lymphomas	Level 3	Phase I/II	[106]
GPI-0100	Sea cucumber (*Holothuria* sp.)	Cancer vaccine adjuvant	Level 3	Phase I	[107]

**Table 3:** Nervous System-Targeting Marine-Derived Analgesic Agents.

Drug	Source	Molecular Structure	Target	Classification of Application Potential	Application
Ziconotide	*Conus magus*	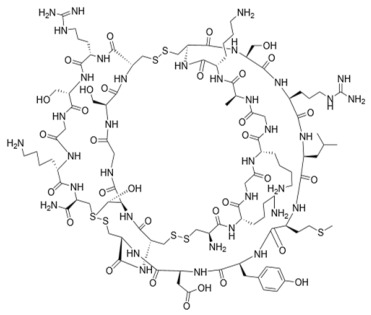	Ca_v_2.2 blocker	Level 2	Intrathecal: refractory chronic neuropathic pain, cancer pain
APETx2	*Anthopleura elgantissima*	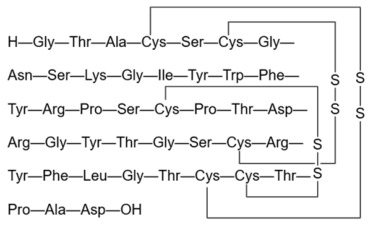	Highly selective ASIC3 inhibitor	Level 2	Preclinical: chemical pain, migraine, bone cancer pain
Tetrodotoxin	Pufferfish, etc.	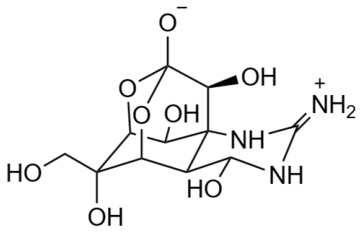	Na_v_1.6/1.7 blocker	Level 2	Clinical phase II/III: neuropathic pain, chemotherapy-induced pain, cancer pain

## Data Availability

No new data were created or analyzed in this study. Data sharing is not applicable to this article.

## Abbreviations

The following abbreviations are used in this manuscript:

ACh	Acetylcholine
ADRB2	Adrenoceptor Beta 2
ALL	Acute Lymphoblastic Leukemia
AML	Acute Myeloid Leukemia
APS8/APS7	Alkylpyridinium Salt 8/7
ASIC3	Acid-Sensing Ion Channel 3
ATP	Adenosine Triphosphate
BDNF	Brain-Derived Neurotrophic Factor
BDS-1	Blood Depressing Substance-1 (a Sea Anemone Toxin)
cAMP	Cyclic Adenosine Monophosphate
Ca_V_2.2	Voltage-Gated Calcium Channel Subtype 2.2 (N-Type Calcium Channel)
CD8^+^ T-cell	Cluster Of Differentiation 8-Positive T Cell
CGRP	Calcitonin Gene-Related Peptide
CHRM1	Cholinergic Receptor Muscarinic 1
CINI	Cancer-Induced Nerve Injury
CIPN	Chemotherapy-Induced Peripheral Neuropathy
CLL	Chronic Lymphocytic Leukemia
CLR	Calcitonin Receptor-Like Receptor
RAMP1	Receptor Activity-Modifying Protein 1
CRPS	Complex Regional Pain Syndrome
eATP	Extracellular Atp
EMT	Epithelial–Mesenchymal Transition
GDNF	Glial Cell-Derived Neurotrophic Factor
GPCRs	G-Protein-Coupled Receptors
HCL	Hairy Cell Leukemia
HNSCC	Head And Neck Squamous Cell Carcinoma
HPLC	High-Performance Liquid Chromatography
IL-18	Interleukin-18
IL-1β	Interleukin-1 Beta
IL-8	Interleukin-8
JAK/STAT	Janus Kinase/Signal Transducer And Activator Of Transcription
K_V_1.x	Voltage-Gated Potassium Channel Subfamily 1.x
MAPK/ERK	Mitogen-Activated Protein Kinase/Extracellular Signal-Regulated Kinase
MDSCs	Myeloid-Derived Suppressor Cells
MMBP	Myeloma bone pain
nAChRs	Nicotinic Acetylcholine Receptors
Na_V_1.7	Voltage-Gated Sodium Channel 1.7
NE	Norepinephrine
NEtD	Neuroendocrine Transdifferentiation
NF-κB	Nuclear Factor Kappa-Light-Chain-Enhancer Of Activated B Cells
NGF	Nerve Growth Factor
NLRP3	Nlr Family Pyrin Domain Containing 3
P2X7	Purinergic Receptor P2X 7
PAR2	Protease-Activated Receptor 2
PD-1	Programmed Cell Death Protein 1
PI3K/AKT/mTOR	Phosphoinositide 3-Kinase/Akt Serine/Threonine Kinase/Mechanistic Target Of Rapamycin
PKA	Protein Kinase A (Camp-Dependent Protein Kinase)
PNI	Perineural Invasion
poly-APS	Polymeric Alkylpyridinium Salts
PSNS	Parasympathetic Nervous System
SCLC	Small Cell Lung Cancer
SNS	Sympathetic Nervous System
STAT3	Signal Transducer And Activator Of Transcription 3
TAMs	Tumor-Associated Macrophages
TME	Tumor Microenvironment
TNBC	Triple-negative breast cancer
TNF-α	Tumor Necrosis Factor Alpha
TRPA1	Transient Receptor Potential Ankyrin 1
TRPV1	Transient Receptor Potential Vanilloid 1
TTX	Tetrodotoxin
VASPI	Visual Analog Scale for Pain Intensity
VGSCs	Voltage-Gated Sodium Channels
